# QiDiTangShen granules alleviates diabetic nephropathy podocyte injury: A network pharmacology study and experimental validation in vivo and vitro

**DOI:** 10.1016/j.heliyon.2023.e23535

**Published:** 2023-12-10

**Authors:** Fei Gao, Ying Zhou, Borui Yu, Huidi Xie, Yang Shi, Xianhui Zhang, Hongfang Liu

**Affiliations:** a Key Laboratory of Chinese Internal Medicine of Ministry of Education and Beijing, Dongzhimen Hospital Affiliated to Beijing University of Chinese Medicine, Beijing, 100700, China; b Department of Endocrinology and Nephrology, Renal Research Institute of Beijing University of Chinese Medicine, Dongzhimen Hospital Affiliated to Beijing University of Chinese Medicine, Beijing, 100700, China; c Department of Nephrology, First Medical Center of Chinese PLA General Hospital, Nephrology Institute of the Chinese People's Liberation Army, State Key Laboratory of Kidney Diseases, National Clinical Research Center for Kidney Diseases, Beijing Key Laboratory of Kidney Disease Research, Beijing, 100853, China; d Health Management Center, Dongzhimen Hospital Affiliated to Beijing University of Chinese Medicine, Beijing, 100700, China

**Keywords:** QiDiTangShen granules, Diabetic nephropathy, Podocyte injury, Network pharmacology, Experimental validation

## Abstract

**Background:**

QiDiTangShen granules (QDTS), a traditional Chinese medicine (TCM) compound prescription, have remarkable efficacy in diabetic nephropathy (DN) patients, and their pharmacological mechanism needs further exploration.

**Methods:**

According to the active ingredients and targets of the QDTS in the TCMSP database, the network pharmacology of QDTS was investigated. The potential active ingredients were chosen based on the oral bioavailability and the drug similarity index. At the same time, targets for DN-related disease were obtained from GeneCards, OMIM, PharmGKB, TTD, and DrugBank. The TCM-component-target network and the protein-protein interaction (PPI) network were constructed with the Cytoscape and STRING platforms, respectively, and then the core targets of DN were selected with CytoNCA. GO and KEGG enrichment analysis using R software. Molecular docking to identify the core targets of QDTS for DN. In vivo, db/db mice were treated as DN models, and the urine microalbuminuria, the pathological changes in the kidney and the protein expression levels of p-PI3K, p-Akt, JUN, nephrin and synaptopodin were detected by immunohistochemistry, immunofluorescence method and Western blotting. After QDTS was used in vitro, the protein expression of mouse podocyte clone-5 (MPC5) cells was detected by immunohistochemistry, immunofluorescence and Western blot.

**Results:**

Through network pharmacology analysis, 153 potential targets for DN in QDTS were identified, 19 of which were significant. The KEGG enrichment analysis indicated that QDTS might have therapeutic effects on IL-17, TNF, AGE-RAGE, PI3K-Akt, HIF-1, and EGFR through interfering with Akt1 and JUN. The main active ingredients in QDTS are *quercetin*, *β-sitosterol*, *stigmasterol* and *kaempferol*. Both in vivo and in vitro studies showed that QDTS could decrease the urine microalbuminuria and renal pathology of db/db mice, and alleviate podocyte injuries through the PI3K/Akt signaling pathway.

**Conclusion:**

Through network pharmacology, in vivo and in vitro experiments, QDTS has been shown to improve the urine microalbuminuria and renal pathology in DN, and to reduce podocyte damage via the PI3K/Akt pathway.

## 1 Introduction

Diabetic nephropathy (DN) is a common complication of type 2 diabetes mellitus and a significant contributor to cardiovascular disease and end-stage kidney disease (ESRD) [1]. Research indicates that approximately 40 % of patients show signs of DN, such as persistent urine microalbuminuria and reduced glomerular filtration rate, within 10 years of being diagnosed with type 2 diabetes mellitus [2]. The impact is even more severe in cases progressing to ESRD, resulting in substantial global economic burdens [3]. Currently, conventional therapies such as renin-angiotensin-aldosterone system blockade, blood glucose control, and weight management have limited effectiveness in achieving satisfactory results [4]. Therefore, there is a need to explore new therapeutic drugs or methods for the treatment of DN.

QiDiTangShen Granules (QDTS), composed of *Astragalus propinquus*, *R. glutinosa, Euryalferox*, *Cornus officinalis*, *Whitmania pigra*, *Rheum officinale* and *Hedyotis diffusa*, have been utilized in the clinical treatment of DN for several years [[5], [6], [7], [8]]. Studies have identified the major constituents of QDTS as formononetin, kaempferol, luteolin, alizarin, emodin, and rhein [9]. Pharmacological research has demonstrated the effectiveness of QDTS and its components in anti-inflammatory, antioxidant, diuretic, immune-enhancing, neuroprotective, and hypoglycemic effects [[9], [10], [11], [12], [13], [14]]. Previous investigations have shown that QDTS significantly reduces urine albumin excretion (UAE) in DN model mice and effectively alleviates renal pathology in db/db mice [15]. Furthermore, QDTS has been found to modulate the composition of intestinal microflora in db/db mice and regulate the bile acid axis within the intestinal microbiota [16]. However, due to the complex nature of traditional Chinese medicine (TCM) with its multiple components and targets, the underlying mechanisms through which QDTS treats DN remain unknown.

TCM formulas are combination of different herbal medicines under the guidance of TCM and contain a variety of small molecule compounds that can jointly regulate disease-specific molecular networks through complex synergistic or antagonistic effects to meet the needs of systemic treatment of complex diseases [17]. Network pharmacology can network, calculate and visualize the various connections between traditional Chinese medicine compounds and diseases. Through basic and deep network analysis, it can systematically reveal the pharmacological mechanism of TCM compounds, and explain the prescription rules of TCM recurrence at the molecular level and overall conditioning effect. Therefore, network pharmacology is widely used in the field of TCM research [18,19].

Considerable evidence suggests that podocyte injury plays a pivotal role in the progression of DN. Morphological and functional impairments of podocytes have been identified as critical factors contributing to glomerular filtration dysfunction and urinary microalbumin [20]. As podocyte cytoskeleton, fissure membrane, and even podocyte loss occur, patients may transition from microalbuminuria to macroalbuminuria [21]. Therefore, preserving podocyte integrity is widely recognized as a crucial aspect of DN management. Previous studies have shown that QDTS reduce urine albumin creatine ratio (UACR) and urine podocin, nephrin and podocalyxin levels in DN patients, suggesting that it can reduce podocyte damage in diabetes [6,8]. Moreover, studies have demonstrated the significant involvement of the PI3K/Akt signaling pathway in podocyte damage during the development of DN [22,23]. As a result, we propose that the PI3K/Akt pathway could be a promising target for DN treatment using QDTS.

**Fig. 1:**
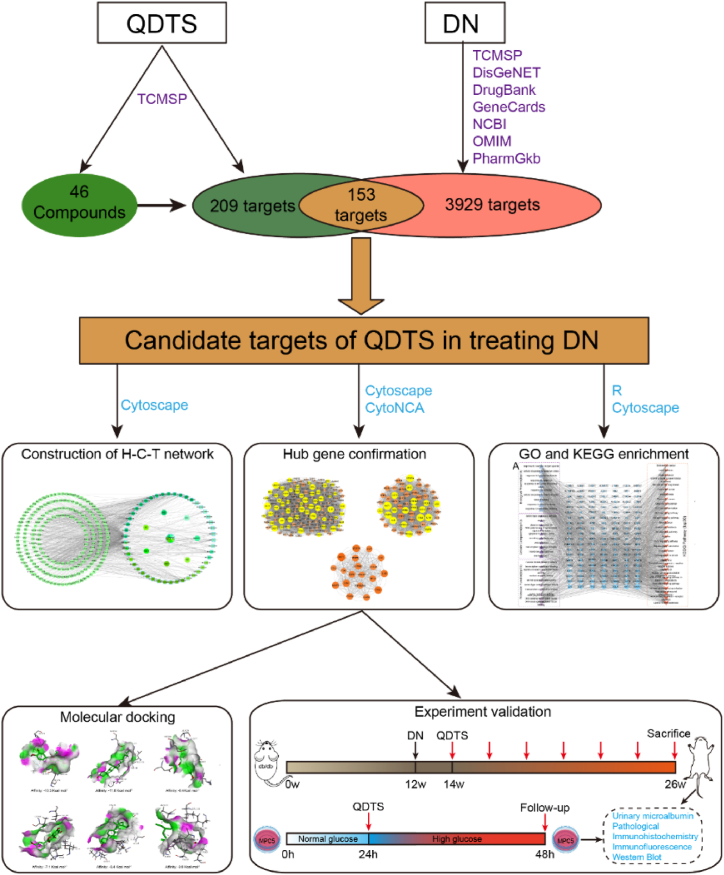
Integrated flowchart of present study.

In this study, the components and targets of QDTS for DN were identified using network pharmacology. Subsequently, the targets were analyzed through protein-protein interaction (PPI) network analysis, as well as Gene Ontology (GO)/Kyoto Encyclopedia of Genes and Genomes (KEGG) functional enrichment analysis. Finally, docking the main components with key targets to verify regulatory relationship and the molecular mechanism underlying the reduction in podocyte damage by QDTS was investigated by regulating the PI3K/Akt signaling pathway in DN mouse models and high glucose-induced podocyte injury models. The flow diagram illustrating the methodology of this study is presented in Fig. 1.

## 2 Methods

### 2.1 Identification of active ingredients and targets in QDTS

All of the active ingredients and targets of QDTS are in the Traditional Chinese Medicine System (TCMSP [24], https://old.tcmspe.com/tcmsp.php, Version.2.3,2020 Annual Update), which contains the chemical ingredients, targets and relationships of TCM. Oral bioavailability (OB) is one of the most important pharmacokinetic parameters in TCM. It reflects the quantity of oral TCM reaching systemic circulation. The drug similarity index (Drug-similarity, DL) is a structural similarity between a compound and a known drug and is widely used to assess the suitability of an ingredient as a drug [24]. Here, OB value ≥ 30 % and DL > 0.18 are the screening criteria for the study. The UniPort database [25] (https://www.uniprot.org, updated in 2022) has been used to translate the full name of the target gene into a gene abbreviation, setting the status to “review” and the species to “human".

### 2.2 Collection of targets for DN

Taking diabetic nephropathy and diabetic kidney disease as keywords, we used the Online Mendelian Inheritance in Man database [26] (OMIN; http://www.omim.org/), the National Center for Biotechnology Information (NCBI, https://www.ncbi.nlm.nih.gov/, grammatical in 2022), Therapeutic Target Database [27] (TTD, https://db.idrblab.org/ttd/), GeneCards [28](https://www.genecards.org/, Version.5.1), DisGeNET [29](https://www.disgenet.org/, Version.7.0), PharmGKB [30](https://www.pharmgkb.org/, updated in 2021) and DrugBank Online [31](https://go.drugbank.com/Magna, version. 5.0) to acquire DN-associated target genes. After the duplicated genes were removed, the rest of the genes were targets of DN related genes. R software Venn packages were used to draw a Venn diagram.

### 2.3 Construction of the ingredient-gene network

Utilizing the R software's Venn package, an analysis was performed to identify potential targets (overlapping genes) of QDTS in the treatment of DN. A Venn diagram was generated to visually represent the results of this analysis. In addition, Cytoscape 3.9.1, an open-source bioinformatics software platform widely employed for complex network analysis and visualization, was utilized. Notably, Cytoscape integrates data pertaining to TCM, active ingredients, and target genes [32].

### 2.4 Construction of the PPI network

To explore PPI, the STRING online platform was employed, allowing retrieval of relevant information. Subsequently, a PPI network was constructed with a focus on “multiprotein” interactions. The resulting nonisolated PPI information was exported as a TSV file and imported into Cytoscape 3.9.1. The network topology of the PPI network was then assessed using the CytoNCA plugin [33]. Key network centrality measures, including degree centrality (DC), betweenness centrality (BC), closeness centrality (CC), eigenvector centrality (EC), network centrality (NC), and local average connectivity-based (LAC), were computed. Nodes with parameter values surpassing the median were identified as significant targets for QDTS treatment in DN. Notably, the size of each node was determined by its degree of connectivity within the network.

### 2.5 GO and KEGG pathway enrichment analysis

For conducting the GO and KEGG enrichment analyses on the overlapping genes, R4.2.0 software was utilized along with the following packages: “clusterProfiler”, “org.Hs.eg.db”, “enrichplot”, and “ggplot2” [34]. The GO functional enrichment analysis encompassed three categories: biological process (BP), molecular function (MF), and cellular component (CC). Among the results obtained, the top 10 biological processes displaying a corrected p value of less than 0.05 were selected. Additionally, the top 30 most enriched pathways were chosen for KEGG analysis.

### 2.6 Molecular docking

The two-dimensional structures of QDTS active components were obtained from the PubChem data library (https://pubchem.ncbi.nlm.nih.gov) as a docking ligand file. Akt1 and JUN protein structure files were downloaded from the PDB online database [35] (https://www.rcsb.org). Molecular docking was carried out by AutoDock VINA software. Discovery Studio software was used to visualize the docking results.

### 2.7 In vivo experiments

Sixteen 8-week-old male db/db mice and eight db/m mice were purchased from Beijing Weitong Lihua Laboratory Animal Technology Co., Ltd [SCXK (Beijing) 2012-0001] and housed in the barrier animal experiment facility of the Key Laboratory of Chinese Medicine at Beijing University of Chinese Medicine (SYXK (Beijing) 2015-0001). Body weight (BW) and fasting blood sugar (FBG) were measured every 2 weeks using an AccuChek OneTouch blood glucose meter (Roche; Basel, Switzerland). Mice with FBG levels consistently greater than 250 mg/dl were considered diabetic models. At 14 weeks of age, the db/db mice were randomly divided into a model group (DN) and a QDTS group (QDTS), with eight mice in each group. The db/m mice were used as the normal control group (NC). The QDTS group was orally administered QDTS granules at a dose of 3.34 g/kg mixed with distilled water, for 12 weeks. The NC and DN groups were treated with the same volume of distilled water through the stomach. The specific composition of QDTS (patent number CN107714817A) has been previously described in detail [9,15,16], and the water extract was supplied by Quzhou Dongle Pharmaceutical Co., Ltd.

The serum was obtained by collecting blood from the orbit and then subsequently centrifuging it at 1500×*g* for 15 min. After euthanizing the mice, the kidneys were swiftly removed and weighed. A small portion of renal tissue was fixed using 2.5 % glutaraldehyde for electron microscopy. Another portion of the sample was fixed with 4 % paraformaldehyde and subjected to staining with hematoxylin and eosin (HE), Masson's trichrome, periodic acid-Schiff-methenamine (PASM), immunohistochemistry (IHC), and immunofluorescence (IF) before being embedded in paraffin. Additionally, a segment of the kidney cortex was rapidly frozen using a liquid nitrogen tank and subsequently stored at −80 °C. This study received approval from the Animal Welfare and Ethics Review Committee of Dongzhimen Hospital [Certificate No.16-14].

### 2.8 In vitro experiments

Conditionally immortalized mouse podocytes (MPC5) were originally obtained from Dr. Jochen Reiser (Rush University Medical Center) and cultured in RPMI 1640 medium (Gibco, New York, USA) supplemented with 10 % fetal bovine serum (Gibco, New York, USA) and 100 U/ml penicillin–streptomycin (Gibco, New York, USA) in the presence of mouse recombinant γ-interferon (γ-INF, Millipore, Billerica, USA) at 33 °C in 5 % CO_2_ for proliferation. Cell differentiation was induced by incubation in RPMI-1640 without IFN-γ at 37 °C with 5 % CO_2_ for 14 days. To investigate the protective effect of QDTS on podocytes, MPC5 cells were randomly assigned to four groups: normal glucose (5.5 mmol/L glucose, N), high glucose (35 mmol/L glucose, H), high glucose + DMSO (35 mmol/L glucose, 200 μg/mL DMSO, DMSO), and high glucose + QDTS (35 mmol/L glucose, 200 μg/mL QDTS). Relevant assays were conducted 48 h after treatment. A detailed description of the preparation and concentration of the QDTS freeze-dried QDTS powder can be found elsewhere [36]. Furthermore, to elucidate the impact of QDTS on the PI3K/Akt pathway in podocytes, QDTS was administered to MCP5 cells along with the PI3K inhibitor LY294002 (20 μmol/L). The expression levels of phosphorylated PI3K, phosphorylated Akt, and downstream molecules including JUN, nephrin, and synaptopodin were assessed.

### 2.9 Determination of urinary microalbumin

Urine microalbuminuria was measured by ELISA (ELISA, Meimian, MM-44399M1, Jiangsu, China). Excretion of albumin in urine (UAER) = urinary microalbuminuria (μg/mL) × urinary volume (mL).

### 2.10 Pathological assay

HE, Masson and PASM staining kits (baso, BA4025, BA4094, BA4079A, Guangzhou, China) were used for 24 h for fixation in 4 % paraformaldehyde (pH 7.4). Meanwhile, the renal cortex was fixed in 2.5 % glutaraldehyde (pH 6.8) at 4 °C for 24 h and then prepared for slicing and analyzed with TEM by an Hmur7650 electron microscope (Hitachi, Tokyo, Japan). The experimental procedure was described in detail in earlier trials [16].

### 2.11 Immunohistochemistry assay

The renal tissue was embedded in paraffin and cut up (4 μm). After dewaxing and hydration, EDTA antigen repair solution (pH 9.0) was used to repair the sections, and then 3 % hydrogen peroxide was used to inhibit the activity of endogenous peroxidase. Then, the goat serum was added to the section for 30 min at 37 °C to prevent nonspecific binding. After incubating with the following primary antibodies: synaptopodin (1: 200; sc-515842; Santa) and nephrin (1: 200; 66970-1-Ig; Proteintech) overnight at 4 °C, PBS was used to wash the sections, and the secondary antibody was incubated at 37 °C for 30 min. After DAB, the nucleus was stained with hematoxylin, dried, cleared(adj) and sealed properly. PBS was substituted for the primary antibody as a negative control. Photos were taken with a Nikon E200 microscope (Nikon, Tokyo, Japan) and a digital camera. Using ImageJ software, the integral optical density of positive regions was calculated, and the protein expression was evaluated.

### 2.12 Immunofluorescence assay

Similar to immunohistochemistry, after dewaxing, hydrating, repairing and incubation in 0.3 % Triton X-100 in PBS for 10 min, the donkey serum was incubated for 30 min, and the first anti-synaptopodin (1: 50, sc-515842, Santa), nephrin (1: 50, 66970-1, Proteintech), phosphorylated PI3K (p-PI3K, 1: 50, YP0765, immunoway) and phosphorylated Akt (p-Akt, 1: 50, YP0006, Immunoway) antibodies were incubated at 4 °C overnight. After being treated on a cover glass placed in 12-well plates, the MPC5 cells were fixed in 4 % paraformaldehyde for 10 min, and then incubated for 10 min with 0.3 % Triton X-100 at room temperature. MPC5 cells were incubated at 4 °C in the primary antibody after blocking with goat serum: synaptopodin (1: 50t, sc-515842Santa) and nephrin (1: 50, 66970-1-Ig, Proteintech). After PBS cleaning, the second antibody was incubated for 1 h at 37 °C with Alexa Fluor 488 or 594, followed by DAPI antifluorescence quenching tablets. Kidney sections and MPC cells were observed and photographed with a Leica laser confocal microscope.

### 2.13 Western blot

RIPA lysis buffer was used to extract total proteins from renal tissues and MPC5 cells. The same amount of protein was loaded onto the SDS PAGE gel after quantitation by BCA. The proteins were then transferred to the PVDF membrane and blocked with 5 % BSA or skim milk Tris buffer saline (TBS). The first antibody was incubated at 4 °C overnight. The secondary antibody was incubated for 1 h at room temperature after washing, and the chemiluminescence (ECL) was enhanced. Using ImageJ software, the integral optical density of the band was analyzed, and the ratio of the IOD to the internal standard β-actin was used as the relative expression.

### 2.14 Statistical analysis

In this study, all data are expressed as the mean ± standard deviation. One-way ANOVA (ANOVA) was used in the statistical software SPSS 25.0. LSD or Dunnett's T3 method was used in pairwise comparisons. GraphPad Prism 8.4.3 software was used to plot the data. The *P* < 0.05 was regarded as a significant difference.

## 3 Results

### 3.1 Bioactive ingredients in QDTS

**Fig. 2:**
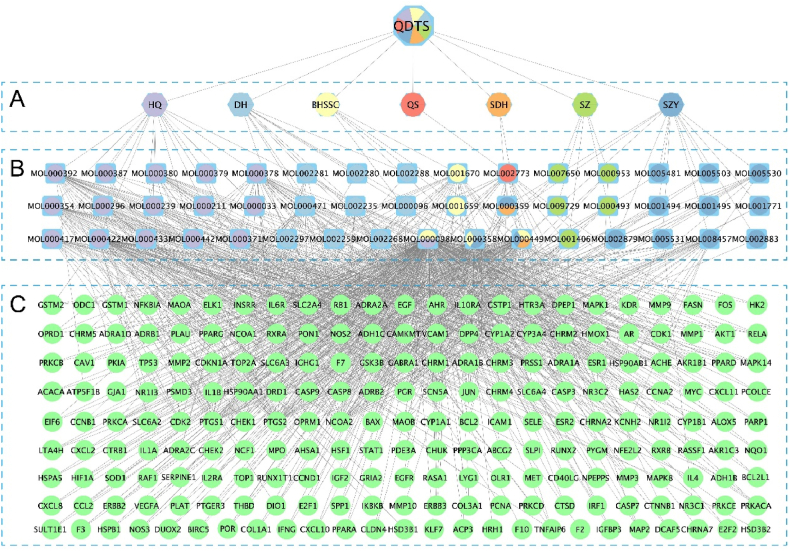
TCM-ingredient-target network of QDTS. The square symbol is the ingredient, the circle is the target. **(A)** Herbs in QDTS. **(B)** Active ingredients. **(C)** Ingredient-related targets. **Abbreviations:** QDTS, QiDiTangShen granules; SDH, *R. glutinosa*; QS, *Euryaleferox*; SZY, *Cornus officinalis*; SZ, *Whitmania pigra*; DH, *Rheum officinale*; BHSSC, *Hedyotis diffusa*.

A total of 46 active ingredients of QDTS were gathered from the TCMSP database based on the screening criteria of OB ≥ 30 % and DL ≥ 0.18. These ingredients include BHSSC 10, DH 10, HQ 16, QS 1, SZY 13, SDH 2, and SZ 5. Notably, BHSSC, HQ, SYR, and SDH share the same composition, while the three herbs have two common ingredients (Supp Table S1). A comprehensive set of 209 ingredient-related targets was compiled. To visualize the TCM-ingredient-target network, Cytoscape was employed to construct the network diagram (Fig. 2).

### 3.2 Ingredient-target network of QDTS and DN

**Fig. 3:**
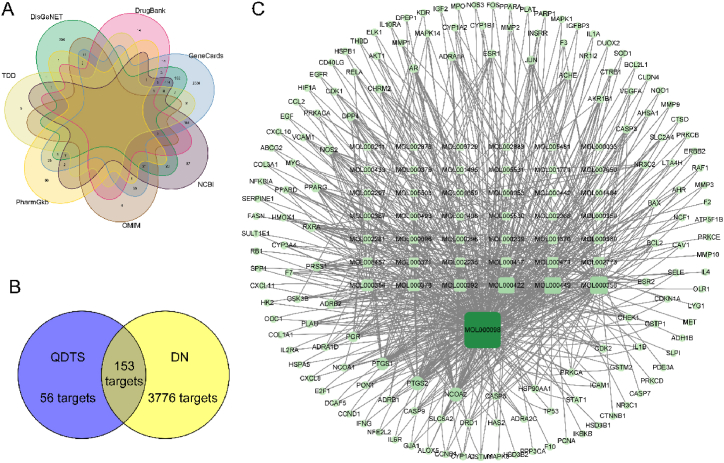
Target of QDTS on DN. **(A)**VENN diagram of targets associated with DN. **(B)**Venn diagram of targets for QDTS and DN related targets. **(C)**Network map of DN treated with QDTS.

A total of 5651 DN-related targets (Fig. 3A, Supp Table S2) were identified from 7 different databases (1189 from DisGeNET, 103 from DrugBank, 3422 from GeneCards, 741 from NCBI, 67 from OMIM, 103 from PharmGkb and 103 from TTD), with 3929 unique targets after removing duplicates. From these, 153 overlapping targets were identified (Fig. 3B, Supp Table S3). Using Cytoscape, a “composite gene network” to identify the major components of QDTS in treating DN was constructed (Fig. 3C). The network topology analysis showed that quercetin had the highest degree of correlation with DN target genes (degree = 216), followed by *β-sitosterol* (degree = 51), *stigmasterol* (degree = 48), and *kaempferol* (degree = 45).

### 3.3 PPI analysis

**Fig. 4:**
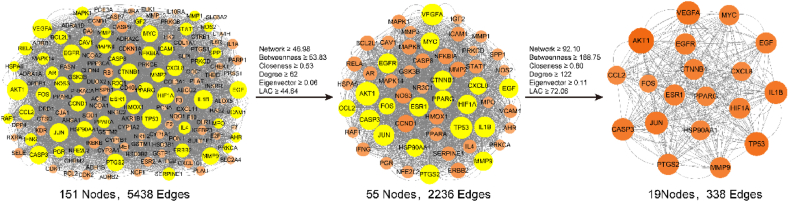
The core genes selected for QDTS in treating DN were identified through network analysis methods.

The data of the 153 overlapping targets were utilized to query the STRING database, enabling the retrieval of information regarding the interactions between the targets and proteins. After removing the nonstructural targets and considering their interactions with other proteins, the resulting STRING data were imported into Cytoscape to construct the PPI network. This network consisted of 151 protein nodes and 5438 edges, representing the interactions between them. Subsequently, CytoNCA was employed to identify the core genes within the PPI network. A total of 19 core genes were screened, including CASP3, HIF1A, VEGFA, ESR1, EGF, MYC, TP53, AKT1, CTNNB1, EGFR, IL1B, FOS, PTGS2, PPARG, CXCL8, JUN, HSP90AA1, CCL2, and MMP9 (Fig. 4).

### 3.4 GO and pathway enrichment analyses

**Fig. 5:**
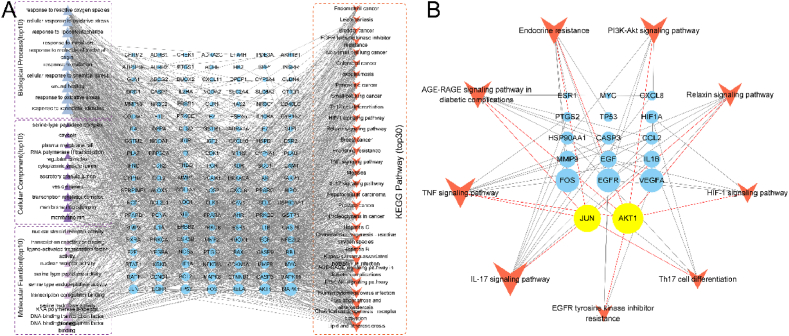
Analysis of Gene Enrichment of DN Target in QDTS. **(A)**Analysis of enrichment of possible objectives. **(B)**Results of QDTS core targets and important signal pathways in DN.

The GO and KEGG pathways were enriched with the 153 overlapping targets using R software. The GO analysis revealed 2286 BP terms, 80 CC terms, and 169 MF terms (Supp Table S4). The top 10 BP, CC, and MF terms are presented (Fig. 5A), which include cell response to chemical stress, oxidative stress reaction, reactive oxygen species reaction, membrane raft, membrane microdomain, plasma membrane raft, DNA-binding transcription factor binding, transcription coregulator binding, and nuclear receptor activity. These results indicate that the process of QDTS in treating DN is multifaceted. The major biological functions include membrane functions, gene transcription regulation, protein translation, and signal transduction.

A total of 181 enrichment pathways were screened using KEGG analysis. Among them, the top 30 most enriched pathways are displayed (Fig. 5A). However, it is noteworthy that many of these pathways are not directly related to DN, such as human cytomegalovirus infection and fluid shear stress and atherosclerosis. To focus on the pathways relevant to DN, a network diagram was constructed incorporating the 19 key targets selected through PPI analysis of QDTS for DN and 9 KEGG enrichment pathways closely associated with DN (Fig. 5B). The network diagram highlighted Akt1 and JUN as the most prominent nodes and emphasized their involvement in significant signaling pathways. Notably, QDTS may exert its effects on DN by modulating Akt1 and JUN and regulating key signaling pathways, such as IL-17, TNF, AGE-RAGE, and PI3K-Akt. This suggests that QDTS might interfere with these pathways to provide therapeutic benefits in DN.

### 3.5 Molecular docking of key ingredients to key targets

**Fig. 6:**
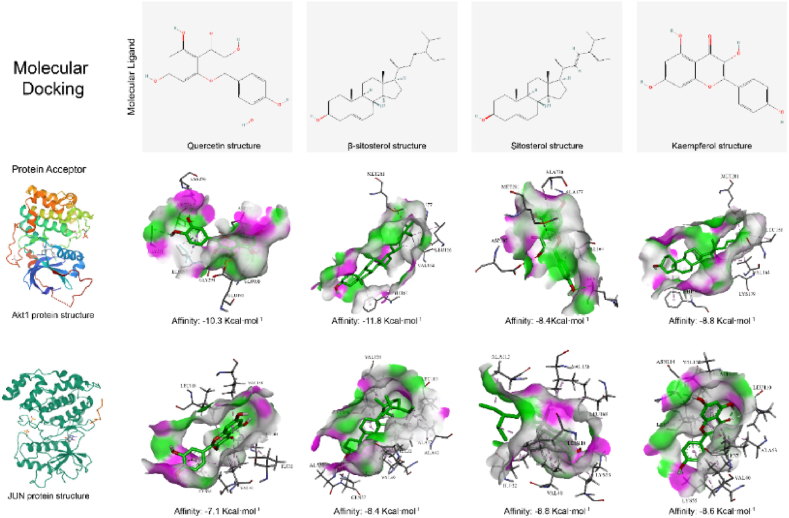
Molecular docking of key ingredients of QDTS to key targets.

To further verify the molecular mechanism of QDTS intervention in DN, quercetin (MOL000098), β-sitosterol (MOL000358), stigmasterol (MOL000449) and kaempferol (MOL000422) in QDTS were regarded as ligands and the key target proteins Akt1 and JUN were regarded as receptors for molecular docking. The results showed that key active components of QDTS had great binding activity to key targets (Fig. 6).

### 3.6 QDTS reduced urinary microalbumin and ameliorated renal pathological damages in db/db mice

**Fig. 7:**
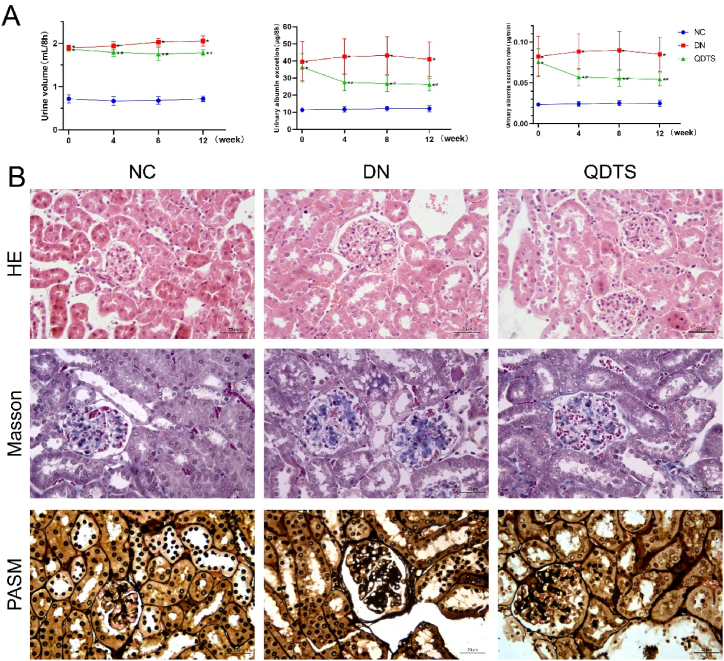
QDTS reduced the level of urine microalbuminuria and improved the renal pathology of db/db mice. **(A)**QDTS reduced the urine volume, urinary microalbumin and UAER level in db/db mice. **(B)**Representative hematoxylin and eosin (HE), Masson, periodic acid-silver methenamine (PASM) staining of kidney sections (magnification 400 × ).

The urinary volume, urine microalbuminuria, and urinary albumin excretion rate (UAER) were significantly increased in the model group compared to the normal control group. However, after 12 weeks of treatment with QDTS, the urine microalbuminuria and UAER were significantly reduced, indicating that QDTS was effective in reducing albuminuria (Fig. 7A). The renal pathology of the model group was more severe than that of the normal control group, with glomerulonephritis, monocyte infiltration, blue collagen fiber deposition, basement membrane thickening, renal tubular epithelium vacuolation, and brush edge exfoliation observed. However, after 12 weeks of treatment with QDTS, these changes were significantly improved, indicating a positive effect of QDTS on DN (Fig. 7B).

### 3.7 QDTS alleviated podocyte injuries in DN

**Fig. 8:**
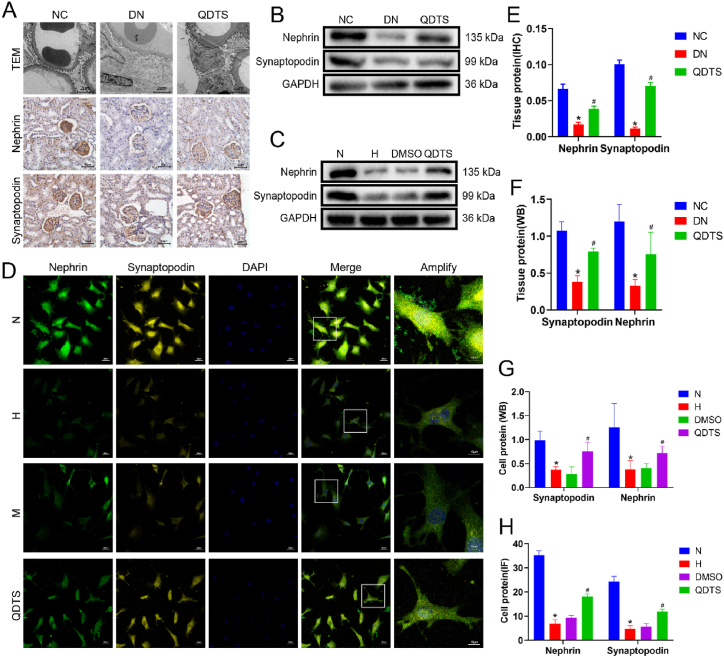
QDTS relieved podocyte damage in DN. **(A)**Observation of Podocyte Injury in db/db mice with TEM (magnification 2000 × ) and Detection the expression of nephrin and synaptopodin by immunohistochemical (magnification 400 × ). **(B)**Detection the expression of nephrin and synaptopodin in db/db mice by Western Blot. **(C)**Expression of nephrin and synaptopodin in MPC5 cells induced by glucose were detected by Western blot. **(D)**Expression of nephrin and synaptopodin in MPC5 cells induced by glucose were determined by immunofluorescence (magnification 400 × ). Semiquantitative analyses of immunostaining**(E)** and Western Blot**(F)** for nephrin and synaptopodin in db/db mice kidney tissue. Semiquantitative analyses of immunostaining**(G)** and Western Blot**(H)** for nephrin and synaptopodin in MPC5 cells. Results were expressed as the mean ± SEM. *P < 0.05 vs NC/N; ^#^*P* < 0.05 vs DN/H. The calculated molecular weight of Nephrin is expected to be around 135 kDa, the actual molecular weight observed is approximately 180 kDa.

Podocyte damage is a significant contributor to albuminuria in patients with DN, and it is often associated with a decrease in podocyte markers such as synaptopodin and nephrin. To assess the ultrastructure of the mouse kidney, transmission electron microscopy was performed. The glomerular basement membrane in the model group was thickened, and fusion between podocyte processes was observed. In contrast, treatment with QDTS resulted in a reduction in podocyte damage in db/db mice (Fig. 8A). These observations suggest that QDTS has a protective effect on podocytes and may contribute to the amelioration of albuminuria in DN.

In both in vivo and in vitro experiments, the expression levels of synaptopodin and nephrin were assessed in the kidney and MPC5 cells. Immunohistochemistry (Fig. 8A), immunofluorescence (Fig. 8D), and Western blot (Fig. 8B and C) techniques were employed for this purpose. The expression of synaptopodin and nephrin in the kidneys of db/db mice was increased by 2.07 times and 2.32 times following treatment with QDTS, as well as increased by 2.01 times and 1.89 times in high glucose-reduced MPC5 cells (Fig. 8E, F, 8G and 8H). This indicates that QDTS has the ability to enhance the expression of synaptopodin and nephrin, thereby reducing podocyte damage in vivo and vitro.

### 3.8 QDTS regulated the PI3K/Akt signaling pathway to modify podocyte injury in DN

**Fig. 9:**
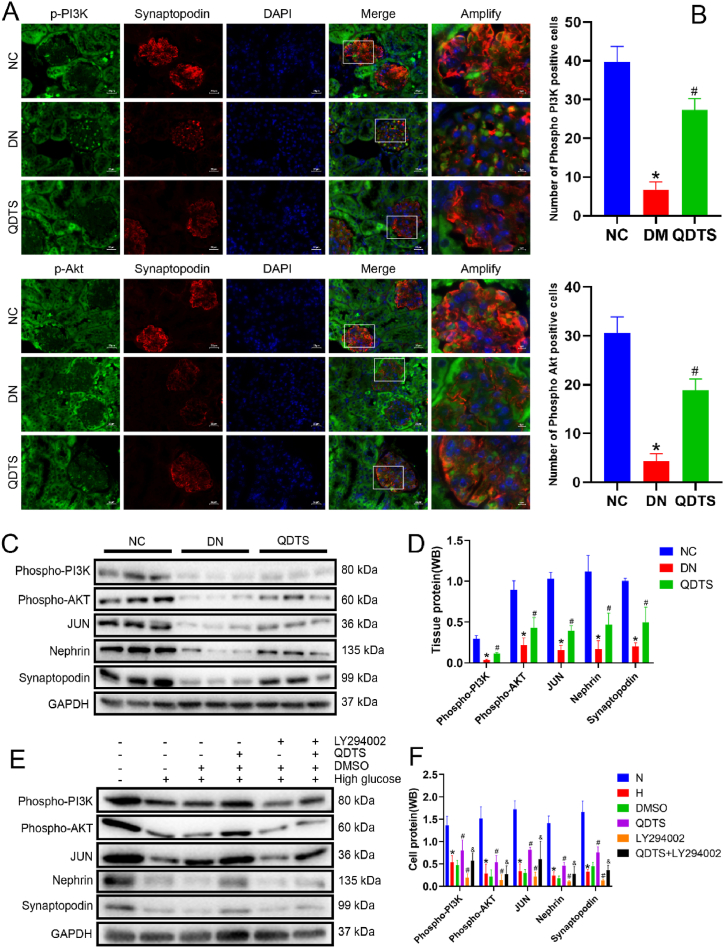
QDTS regulates the pathway of PI3K/Akt to modify podocyte damage in DN. **(A)**The expression of p-PI3K and p-Akt in renal podocytes of mice was studied with immunofluorescence (magnification 400 × ). **(B)**Semiquantitative analyses of immunofluorescence for p-PI3K and p-Akt in db/db mice glomerular. **(C)**Western blot was used to detect p-PI3K, p-Akt, JUN, nephrin and synaptopodin in the kidney of mice. **(D)**Semiquantitative analyses of Western blot in kidney tissue. **(E)**Western blot was used to detect p-PI3K, p-Akt, JUN, nephrin and synaptopodin in MPC5 cells. **(F)**Semiquantitative analyses of Western blot in MPC5 cells. Results were expressed as the mean ± SEM. *P < 0.05 vs NC/N; ^#^*P* < 0.05 vs DN/H; ^&^*P* < 0.05 vs LY294002.

In vivo, the expression levels of p-PI3K and p-Akt in the PI3K/Akt pathway were investigated to observe the inhibitory effect of PI3K/Akt on podocytes. Immunofluorescence analysis (Fig. 9A and B) showed a significant decrease in the expression of p-PI3K and p-Akt in the model group compared to the control group. This trend was also observed in the Western blot analysis that p-PI3K was decreased by 11.54 %, p-Akt was decreased by 24.19 %, synaptopodin was decreased by 19.82 %, and nephrin was decreased by 15.09 % (Fig. 9C and D), indicating the inhibition of the PI3K/Akt pathway in DN. However, treatment with QDTS resulted in an upregulation of p-PI3K (3.32 times) and p-Akt (1.99 times) in the podocytes of DN mice, as shown by immunofluorescence and Western blot analyses. Furthermore, Western blot analysis demonstrated that QDTS treatment increased the expression of JUN (2.54 times), nephrin (2.77 times), and synaptopodin (2.49 times) in podocytes (Fig. 9C and D).

In vitro, the expression of p-PI3K and p-Akt in MPC5 cells treated with HG, QDTS (200 μM) and LY294002 (a PI3K inhibitor) was determined. DMSO was used as a vehicle control group and had no effect on its expression. Western blot analysis revealed similar changes in MPC5 cells induced by high glucose (Fig. 9E and F), suggesting the inhibition of PI3K/Akt and downstream expression of JUN (2.41 times), nephrin (1.97 times) and synaptopodin (2.31 times) could be reversed by QDTS.

QDTS was found to upregulate the expression of p-PI3K and p-Akt in podocytes both in vivo and in vitro. Inhibiting the PI3K/Akt pathway in vitro with LY294002 led to a decrease in the expression of p-PI3K (36.12 %) and p-Akt(48.57 %), as well as the downstream targets JUN(64.96 %), Nephrin(45.61 %), and Synaptopodin(40.17 %). However, treatment with QDTS and LY294002 was able to suppress this downregulation activity and increase the expression of JUN (2.75 times), nephrin (2.60 times), and synaptopodin (2.76 times) (Fig. 9E and F). This suggests that QDTS can inhibit high glucose-induced podocyte injury through the PI3K/Akt pathway.

## 4 Discussion

In this study, a new mechanism of action for QDTS in reducing DN through a combination of network pharmacology and experimental validation was discovered. Network pharmacology analysis identified the active components of QDTS, with quercetin being the most prominent component, followed by β-sitosterol, stigmasterol, and kaempferol. These findings were consistent with the results of prior UHPLC-MS analysis of QDTS extracts [9]. Quercetin, kaempferol, and other active components of QDTS have been extensively studied and shown to have various beneficial effects in DN animal models [37,38]. These effects include antioxidation, anti-inflammation, anti-fibrosis, regulation of renal lipid accumulation, promotion of podocyte autophagy, and inhibition of the overactivity of the renin-angiotensin-aldosterone system (RAAS) [39]. These mechanisms contribute to the improvement of kidney function and alleviation of DN. Furthermore, emodin, rhein, and crocetin, which are also present in QDTS, have been reported to have beneficial effects in DN, such as enhancing the expression of transforming growth factor-beta 1 (TGF-β1), reducing renal fibrosis, regulating metabolism, and mitigating oxidative stress [[40], [41], [42]]. Overall, the experimental findings validated the predictions made through network pharmacology analysis and supported the potential of QDTS in reducing DN through multiple mechanisms, including antioxidation, anti-inflammation, anti-fibrosis, regulation of lipid metabolism, promotion of podocyte autophagy, and modulation of the RAAS pathway.

The PPI network analysis of the QDTS-DN overlapping targets revealed significant interactions among various proteins, with a particular emphasis on key proteins such as AKT1, TP53, VEGFA, IL1B, JUN, CASP3, EGFR, and PTGS2. These proteins were found to play important roles in the network, and their interactions were observed to be closely connected. This suggests that these proteins may be critical mediators of QDTS's therapeutic effects in DN. To gain further insights into the biological functions of the overlapping targets, GO enrichment analysis was conducted. The analysis revealed that the biological processes associated with the overlapping genes were highly enriched in oxidative stress. This indicates that QDTS may exert its effects by targeting oxidative stress pathways in DN, which is consistent with its potential antioxidative properties. Oxidative stress is known to contribute to the development and progression of DN, and by targeting this pathway, QDTS may help alleviate the oxidative stress burden in DN patients. Additionally, the analysis showed that the cell components associated with the overlapping genes were highly enriched in membrane-related structures. This suggests that the target genes affected by QDTS in DN may primarily act on cellular membranes to regulate various biological processes, including gene transcription, protein translation, and signal transduction. Membranes play a crucial role in cellular function and signaling, and the enrichment of membrane-related components indicates that QDTS may modulate these processes through membrane-associated mechanisms.

DN is characterized by membranous structural lesions, including thickening of the glomerular basement membrane and enlargement of the mesangial area [43]. These changes are associated with podocyte damage, which plays a crucial role in the development of DN. The glomerular filtration barrier, composed of podocytes, glomerular basement membrane, endothelial cells, and mesangial cells, regulates the filtration of substances in the kidney [44,45]. When podocytes are damaged, it can lead to increased permeability and leakage of albumin, resulting in albuminuria [20]. The results indicate that treatment with QDTS significantly reduces renal pathological damage in db/db mice. Specifically, QDTS was found to alleviate glomerular basement membrane thickening, reduce mesangial matrix proliferation, and mitigate podocyte damage. Moreover, QDTS treatment resulted in a reduction in urinary microalbumin levels in db/db mice.

Podocytes play a crucial role in maintaining the integrity of the glomerular filtration barrier in the kidney. They are attached to the glomerular basement membrane through α3β1 integrin and form specialized foot processes, which contribute to the formation of the filtration barrier [20]. Nephrin, a transmembrane protein, is located on the podocyte's foot processes and is essential for the structure and function of the filtration barrier. It forms complexes with other proteins such as podocin and CD2AP, which are critical for maintaining the integrity of the slit membrane [20]. Synaptopodin, an actin-binding protein, is important for regulating the podocyte cytoskeleton and maintaining its structural integrity [45]. Both nephrin and synaptopodin are essential for the normal function of podocytes and the prevention of protein loss in urine. In the early stages of DN, the expression of nephrin and synaptopodin is often reduced, indicating podocyte injury. Immunohistochemical analysis in this study demonstrated that nephrin was specifically expressed in the glomerulus of mice. The expression of nephrin and synaptopodin was decreased in the model group, indicating podocyte damage. However, treatment with QDTS resulted in higher expression levels of nephrin and synaptopodin, both in the kidney and in MPC5 cells cultured with high glucose. These findings further confirm the protective effect of QDTS on podocytes and suggest its potential in preserving the integrity of the filtration barrier and preventing podocyte injury in DN.

The KEGG pathway enrichment analysis of the overlapping targets revealed a total of 181 pathways that may be involved in the regulation of podocyte injury in DN. To further understand the upstream regulation of podocyte injury, a network diagram was constructed, linking the 19 key targets of QDTS for DN selected by PPI with 9 KEGG enrichment pathways closely associated with DN. In this network diagram, Akt1 and JUN were found to have the largest nodes, indicating their significant roles in the regulatory network. Two targets were also involved in multiple enriched pathways, suggesting their potential as key regulators in DN. Based on these findings, it can be speculated that QDTS may primarily exert its therapeutic effects in DN by interfering with Akt1 and JUN. These targets may regulate various signaling pathways, including IL-17 [46], TNF [47], AGE-RAGE [48], PI3K-Akt [49], HIF-1 [50], and EGFR [51], which are known to be involved in the pathogenesis of DN. By targeting key signaling pathways, QDTS may modulate processes such as inflammation, oxidative stress, and cellular proliferation, ultimately leading to the amelioration of podocyte injury and the progression of DN. It is important to note that further experimental studies are needed to validate the specific mechanisms of action and therapeutic potential of QDTS in targeting these signaling pathways and protecting podocytes in DN.

**Fig. 10:**
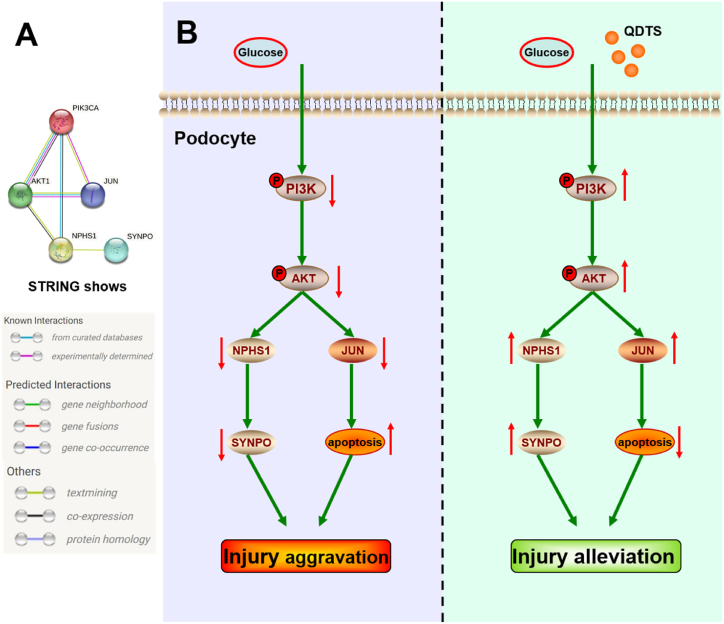
Schematic mechanism of QDTS regulating PI3K/AKT pathway to reduce podocyte damage in diabetes nephropathy. **(A)** The STRING database shows that there are interactions between PI3K, Akt, JUN, Synaptopodin, and Nephrin. **(B)**Model of regulation of QDTS on p-PI3K, p-Akt, JUN, Synaptopodin, and Nephrin in podocyte of DN. **Abbreviations:** PIK3CA, PI3K; NPHS1, Synaptopodin; SYNPO, Nephrin.

Akt1, a crucial effector of the PI3K/Akt signaling pathway [52], plays a significant role in podocyte injury [53], extracellular matrix accumulation [54], mesangial cell proliferation and renal tubular epithelial-mesenchymal transition [55,56]. Similarly, JUN, a downstream regulatory protein of the PI3K/Akt pathway [57], is involved in the regulation of autophagy and apoptosis [58,59]. The expression of p-PI3K and p-Akt in the PI3K/Akt pathway indicates that PI3K/Akt signaling is inhibited in the context of DN. However, QDTS treatment upregulates the expression of p-PI3K and p-Akt in podocytes of DN mice, both in vivo and in vitro. The results obtained in MPC5 cells treated with a PI3K inhibitor (LY294002) and high glucose further confirm the inhibitory effect of QDTS on high glucose-induced downregulation of p-PI3K and p-Akt. Coculturing high glucose-treated podocytes with QDTS and LY294002 inhibited the downregulation effect observed in high glucose-treated cells alone (Fig. 9). Moreover, the expression of JUN, nephrin, and synaptopodin, downstream targets of the PI3K/Akt pathway, increased upon QDTS treatment, supporting the role of QDTS in inhibiting podocyte injury through the PI3K/Akt pathway (Fig. 10A and B).

Additionally, studies have shown that the accumulation of advanced glycation end products (AGEs) in a high glucose environment is a major pathogenic factor in DN [48]. Activation of the PI3K/Akt pathway is observed in the kidneys of diabetic patients upon the interaction of AGEs with the receptor for AGEs (RAGE), resulting in a complex microenvironment that exacerbates oxidative stress, inflammation, and apoptosis, ultimately leading to damage to kidney cells, including podocytes and epithelial cells [48,60]. In line with these findings, QDTS has been shown to downregulate the expression of AGEs and RAGE proteins in the kidneys of db/db mice, corroborating the KEGG enrichment results of this study [61]. These findings highlight the multimodal features of QDTS in the treatment of DN.

There are some limitations in this present study. Although the PI3K/Akt is a key signaling pathway for QDTS in the treatment of DN through network pharmacology and experimental verification, it needs further experiment to confirm that QDTS reduces podocyte damage in diabetic nephropathy by targeting AKT1 and JUN, such as gene intervention. Meanwhile, AKT1 is involved in podocyte damage, but its related molecular mechanisms are still relatively lacking. In addition, quercetin and sitosterol may be important effective ingredients for QDTS in treatment for DN. In subsequent experiments, we will focus on which compounds in QDTS actually exert therapeutic effects.

## 5 Conclusion

In conclusion, this study provides a comprehensive understanding of the molecular mechanisms underlying the therapeutic effects of QDTS in DN. This highlights the role of the PI3K/Akt signaling pathway in mediating the podocyte protective effects of QDTS, which contributes to the overall amelioration of proteinuria and renal pathological damage in DN. These findings provide valuable insights into the specific molecular mechanisms of QDTS in the treatment of DN and pave the way for future research and clinical applications.

## Data availability statement

Data included in article/supp. material/referenced in article.

## Availability of data and materials

The datasets used during the present study are available from the corresponding author on reasonable request.

## Consent for publication

All authors approved the publication of this manuscript.

## Funding

This work was supported by the 10.13039/501100001809National Natural Science Foundation of China (No.81774273).

## Additional information

No additional information is available for this paper.

## CRediT authorship contribution statement

**Fei Gao:** Writing – original draft, Validation, Data curation. **Ying Zhou:** Writing – original draft, Validation. **Borui Yu:** Validation. **Huidi Xie:** Formal analysis, Data curation. **Yang Shi:** Validation. **Xianhui Zhang:** Writing – review & editing, Supervision, Methodology. **Hongfang Liu:** Writing – review & editing, Supervision, Methodology, Funding acquisition.

## Declaration of competing interest

The authors declare the following financial interests/personal relationships which may be considered as potential competing interests: Liu hongfang reports financial support was provided by 10.13039/501100001809National Natural Science Foundation of China. Liu hongfang has patent #CN107714817A issued to liu hongfang.

## Abbreviations

QDTS: QiDiTangShen Granules
DN: Diabetic Nephropathy
TCM: Traditional Chinese Medicine
MPC5: Mouse Podocyte Clone-5
ESRD: End Stage Kidney Disease
UAE: Urine Albumin Excretion
TCMSP: Traditional Chinese Medicine Systems Pharmacology Database and Analysis Platform
OB: Oral Bioavailability
DL: Drug Similarity
GeneCards: Human Gene Database
HE: Hematoxylin and Eosin
Masson: Masson's trichrome
PASM: Periodic Acid Schiff Methenamine
IHC: Immunohistochemistry
IF: Immunofluorescence
DC: Degree Centrality
BC: Betweenness Centrality
CC: Closeness Centrality
EC: Eigenvector Centrality
NC: Network Centrality
LAC: Local Average Connectivity-based
BP: Biological Process
MF: Molecular Function
CC: Cell Component
GO: Gene Ontology
KEGG: Kyoto Encyclopedia of Genes and Genomes
BW: Body Weight
FBG: Fasting Blood Sugar
NPHS1: Synaptopodin
SDH: *R.glutinosa*
QS: *Euryaleferox*
SZY: *Cornus officinalis*
SZ: *Whitmania pigra*
DH: *Rheum officinale*
BHSSC: *Hedyotis diffusa*
DMSO: Dimethyl Sulfoxide
UAER: Urine albumin Excretion Rate
AGEs: Advanced Glycation End Products
RAGE: Receptor for advanced glycation end products
RAAS: Renin Angiotensin Aldosterone System
TGF-β1: Transforming Growth Factor beta 1
PIK3CA: PI3K
NPHS1: Synaptopodin
SDH: *R.glutinosa*
QS: *Euryaleferox*
SZY: *Cornus officinalis*
SZ: *Whitmania pigra*
DH: *Rheum officinale*
BHSSC: *Hedyotis diffusa*
SYNPO: Nephrin

## References

[1] Thipsawat S. (2021). Early detection of diabetic nephropathy in patient with type 2 diabetes mellitus: a review of the literature. Diabetes Vasc. Dis. Res..

[2] Alicic R.Z., Rooney M.T., Tuttle K.R. (2017). Diabetic kidney disease: challenges, progress, and possibilities. Clin. J. Am. Soc. Nephrol..

[3] Selby N.M., Taal M.W. (2020). An updated overview of diabetic nephropathy: diagnosis, prognosis, treatment goals and latest guidelines. Diabetes Obes. Metabol..

[4] Mende C.W. (2022). Chronic kidney disease and SGLT2 inhibitors: a review of the evolving treatment landscape. Adv. Ther..

[5] Han Y.L., Liu H.F., Lou X.E. (2014). Treatment of stage Ⅳ proteinuria in diabetes nephropathy of deficiency of both qi and yin with combination of disease and syndrome. J. Changchun Univ. Tradit. Chin. Med..

[6] Lian X.Y., Liu H.F., Miao G.Z. (2022). Qidi Tangshen Granule in the treatment of proteinuria in patients with diabetes and kidney disease and its effect on urinary fissure and podocyte. J. Beijing Univ. Tradit. Chin. Med..

[7] Wang D., Xu J., Li X. (2020). Effect of qiditangshen recipe on renal protection in patients with diabetes nephropathy stage Ⅲ. J. Beijing Univ. Tradit. Chin. Med..

[8] Xu J., Liu H.F., Miao G.Z. (2020). Treatment of DKD microalbuminuria with qidi tangshen formula and its effect on urinary PCX and LAP. J. Tianjin Univ. Tradit. Chin. Med..

[9] Wang X., Zhao L., Ajay A.K. (2019). QiDiTangShen granules activate renal nutrient-sensing associated autophagy in db/db mice. Front. Physiol..

[10] Gao X., Liu Y., An Z. (2021). Active components and pharmacological effects of Cornus officinalis: literature review. Front. Pharmacol..

[11] Wang M., Ke Y., Li Y. (2021). The nephroprotective effects and mechanisms of rehmapicrogenin include ROS inhibition via an oestrogen-like pathway both in vivo and in vitro. Biomed. Pharmacother..

[12] Zhang R., Ma C., Wei Y. (2021). Isolation, purification, structural characteristics, pharmacological activities, and combined action of Hedyotis diffusa polysaccharides: a review. Int. J. Biol. Macromol..

[13] Zhang Z.Y., Ma N., Tao L.J. (2019). Linear peptides containing d-leucine with neuroprotective activities from the leech Whitmania pigra whitman. J. Nat. Prod..

[14] Zhu X.M., Liu X.Y., Xia C.G. (2021). Effects of dietary Astragalus Propinquus Schischkin polysaccharides on growth performance, immunological parameters, antioxidants responses and inflammation-related gene expression in Channa argus. Comp. Biochem. Physiol. C Toxicol. Pharmacol..

[15] Gao X., Liu H., An Z. (2018). QiDiTangShen granules reduced diabetic kidney injury by regulating the phosphorylation balance of the tyrosine and serine residues of insulin receptor substrate 1. Evid. Based Complement Alternat. Med..

[16] Wei H., Wang L., An Z. (2021). QiDiTangShen granules modulated the gut microbiome composition and improved bile acid pro fi les in a mouse model of diabetic nephropathy. Biomed. Pharmacother..

[17] Li S., Zhang B. (2013). Traditional Chinese medicine network pharmacology: theory, methodology and application. Chin. J. Nat. Med..

[18] Li H., Zhao L., Zhang B. (2014). A network pharmacology approach to determine active compounds and action mechanisms of ge-gen-qin-lian decoction for treatment of type 2 diabetes. Evid. Based Complement Alternat. Med..

[19] Luo T.T., Lu Y., Yan S.K. (2020). Network pharmacology in research of Chinese medicine formula: methodology, application and prospective. Chin. J. Integr. Med..

[20] Cao X., Wei R., Zhou J. (2019). Wenshen Jianpi recipe, a blended traditional Chinese medicine, ameliorates proteinuria and renal injury in a rat model of diabetic nephropathy. BMC Compl. Alternative Med..

[21] Tang G., Li S., Zhang C. (2021). Clinical efficacies, underlying mechanisms and molecular targets of Chinese medicines for diabetic nephropathy treatment and management. Acta Pharm. Sin. B.

[22] Sun Y., Deng M., Ke X. (2021). Epidermal growth factor protects against high glucose-induced podocyte injury possibly via modulation of autophagy and PI3K/AKT/mTOR signaling pathway through DNA methylation. Diabetes Metab. Syndr. Obes..

[23] Zhang Y., Wang Y., Luo M. (2019). Elabela protects against podocyte injury in mice with streptozocin-induced diabetes by associating with the PI3K/Akt/mTOR pathway. Peptides.

[24] Ru J., Li P., Wang J. (2014). TCMSP: a database of systems pharmacology for drug discovery from herbal medicines. J. Cheminf..

[25] Wang Y., Wang Q., Huang H. (2021). A crowdsourcing open platform for literature curation in UniProt. PLoS Biol..

[26] Lupski J.R. (2021). Clan genomics: from OMIM phenotypic traits to genes and biology. Am. J. Med. Genet..

[27] Zhou Y., Zhang Y., Zhao D. (2023). TTD: therapeutic Target Database describing target druggability information. Nucleic Acids Res..

[28] Safran M., Dalah I., Alexander J. (2010).

[29] Pinero J., Ramirez-Anguita J.M., Sauch-Pitarch J. (2020). The DisGeNET knowledge platform for disease genomics: 2019 update. Nucleic Acids Res..

[30] Whirl-Carrillo M., Huddart R., Gong L. (2021). An evidence-based framework for evaluating pharmacogenomics knowledge for personalized medicine. Clin. Pharmacol. Ther..

[31] Wishart D.S., Feunang Y.D., Guo A.C. (2018). DrugBank 5.0: a major update to the DrugBank database for 2018. Nucleic Acids Res..

[32] Shannon P., Markiel A., Ozier O. (2003). Cytoscape: a software environment for integrated models of biomolecular interaction networks. Genome Res..

[33] Tang Y., Li M., Wang J. (2015). CytoNCA: a cytoscape plugin for centrality analysis and evaluation of protein interaction networks. Biosystems.

[34] Shi H., Hou Y., Su X. (2022). Mechanism of action of Tripterygium wilfordii for treatment of idiopathic membranous nephropathy based on network pharmacology. Ren. Fail..

[35] Burley S.K., Berman H.M., Kleywegt G.J. (2017). Protein data bank (PDB): the single global macromolecular structure archive. Methods Mol. Biol..

[36] Wang L., Zhao L., Wei H.L. (2021). Effect of qidi tangshen formula on the expression of autophagy related factors in podocytes induced by high glucose. Chin. J. Gerontology.

[37] Hu T., Yue J., Tang Q. (2022). The effect of quercetin on diabetic nephropathy (DN): a systematic review and meta-analysis of animal studies. Food Funct..

[38] Li Z., Deng H., Guo X. (2022). Effective dose/duration of natural flavonoid quercetin for treatment of diabetic nephropathy: a systematic review and meta-analysis of rodent data. Phytomedicine.

[39] Hu Q., Qu C., Xiao X. (2021). Flavonoids on diabetic nephropathy: advances and therapeutic opportunities. Chin. Med..

[40] Hu H.C., Zheng L.T., Yin H.Y. (2019). A significant association between rhein and diabetic nephropathy in animals: a systematic review and meta-analysis. Front. Pharmacol..

[41] Yang X. (2019). Design and optimization of crocetin loaded PLGA nanoparticles against diabetic nephropathy via suppression of inflammatory biomarkers: a formulation approach to preclinical study. Drug Deliv..

[42] Zeng J.Y., Wang Y., Miao M. (2021). The effects of rhubarb for the treatment of diabetic nephropathy in animals: a systematic review and meta-analysis. Front. Pharmacol..

[43] Thomas M.C. (2021). Targeting the pathobiology of diabetic kidney disease. Adv. Chron. Kidney Dis..

[44] Marshall C.B. (2016). Rethinking glomerular basement membrane thickening in diabetic nephropathy: adaptive or pathogenic?. Am. J. Physiol. Ren. Physiol..

[45] Tung C.W., Hsu Y.C., Shih Y.H. (2018). Glomerular mesangial cell and podocyte injuries in diabetic nephropathy. Nephrology.

[46] Ettou S., Jung Y.L., Miyoshi T. (2020). Epigenetic transcriptional reprogramming by WT1 mediates a repair response during podocyte injury. Sci. Adv..

[47] Li G., Zhang J., Liu D. (2021). Identification of hub genes and potential ceRNA networks of diabetic nephropathy by weighted gene Co-expression network analysis. Front. Genet..

[48] Sanajou D., Ghorbani Haghjo A., Argani H. (2018). AGE-RAGE axis blockade in diabetic nephropathy: current status and future directions. Eur. J. Pharmacol..

[49] Su W.Y., Li Y., Chen X. (2021). Ginsenoside Rh1 improves type 2 diabetic nephropathy through AMPK/PI3K/Akt-Mediated inflammation and apoptosis signaling pathway. Am. J. Chin. Med..

[50] Cheng L., Qiu X., He L. (2022). MicroRNA-122-5p ameliorates tubular injury in diabetic nephropathy via FIH-1/HIF-1alpha pathway. Ren. Fail..

[51] Li Y., Pan Y., Cao S. (2021). Podocyte EGFR inhibits autophagy through upregulation of rubicon in type 2 diabetic nephropathy. Diabetes.

[52] Huang X., Liu G., Guo J. (2018). The PI3K/AKT pathway in obesity and type 2 diabetes. Int. J. Biol. Sci..

[53] Chiou T.T., Chau Y.Y., Chen J.B. (2021). Rapamycin attenuates PLA2R activation-mediated podocyte apoptosis via the PI3K/AKT/mTOR pathway. Biomed. Pharmacother..

[54] Wang J., Zhu H., Huang L. (2019). Nrf2 signaling attenuates epithelial-to-mesenchymal transition and renal interstitial fibrosis via PI3K/Akt signaling pathways. Exp. Mol. Pathol..

[55] Qian X., He L., Hao M. (2021). YAP mediates the interaction between the Hippo and PI3K/Akt pathways in mesangial cell proliferation in diabetic nephropathy. Acta Diabetol..

[56] Zhang Z., Wu W., Fang X. (2020). Sox9 promotes renal tubular epithelial-mesenchymal transition and extracellular matrix aggregation via the PI3K/AKT signaling pathway. Mol. Med. Rep..

[57] Zhu X., Jia X., Cheng F. (2020). c-Jun acts downstream of PI3K/AKT signaling to mediate the effect of leptin on methionine adenosyltransferase 2B in hepatic stellate cells in vitro and in vivo. J. Pathol..

[58] De Borst M.H., Prakash J., Melenhorst W.B. (2007). Glomerular and tubular induction of the transcription factor c-Jun in human renal disease. J. Pathol..

[59] Ma F.Y., Flanc R.S., Tesch G.H. (2007). A pathogenic role for c-Jun amino-terminal kinase signaling in renal fibrosis and tubular cell apoptosis. J. Am. Soc. Nephrol..

[60] Xiao Y., Liu Y., Lai Z. (2021). An integrated network pharmacology and transcriptomic method to explore the mechanism of the total Rhizoma Coptidis alkaloids in improving diabetic nephropathy. J. Ethnopharmacol..

[61] Yu B.R. (2020).

