# Supplementary material for: QiDiTangShen granules alleviates diabetic nephropathy podocyte injury: A network pharmacology study and experimental validation in vivo and vitro
Source: Heliyon. 2023 Dec 10;10(1):e23535. doi: 10.1016/j.heliyon.2023.e23535 (PMC10784173; doi:10.1016/j.heliyon.2023.e23535)

Figure S1(8B)

mice kidney tissue
Nephrin(135 kDa)

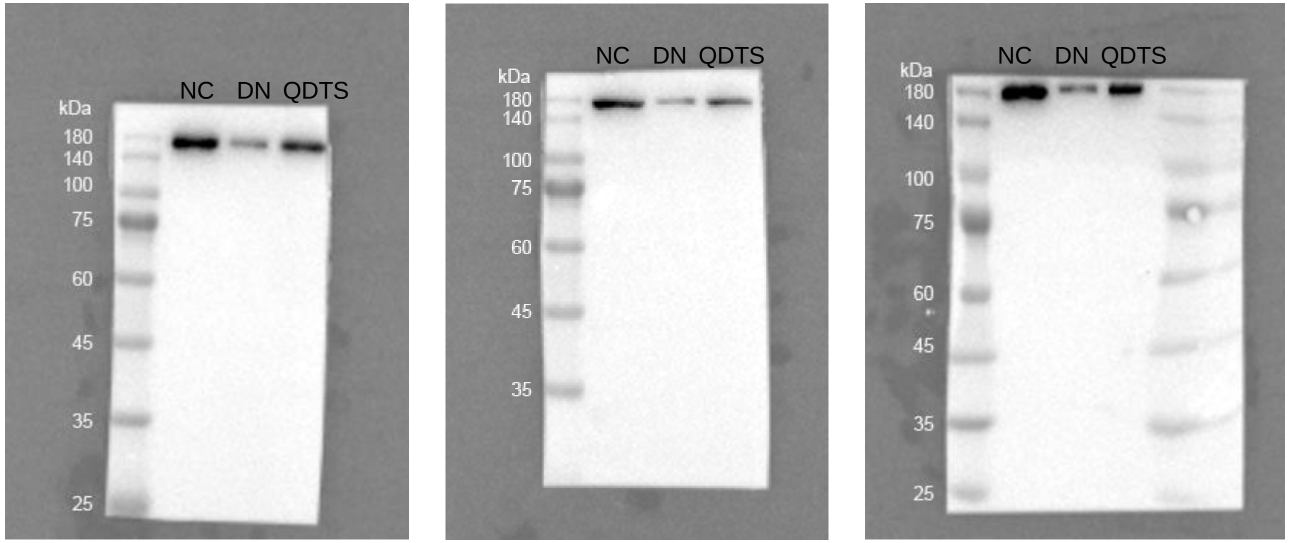

Figure S2(8B)

mice kidney tissue
Synaptopodin(99 kDa)

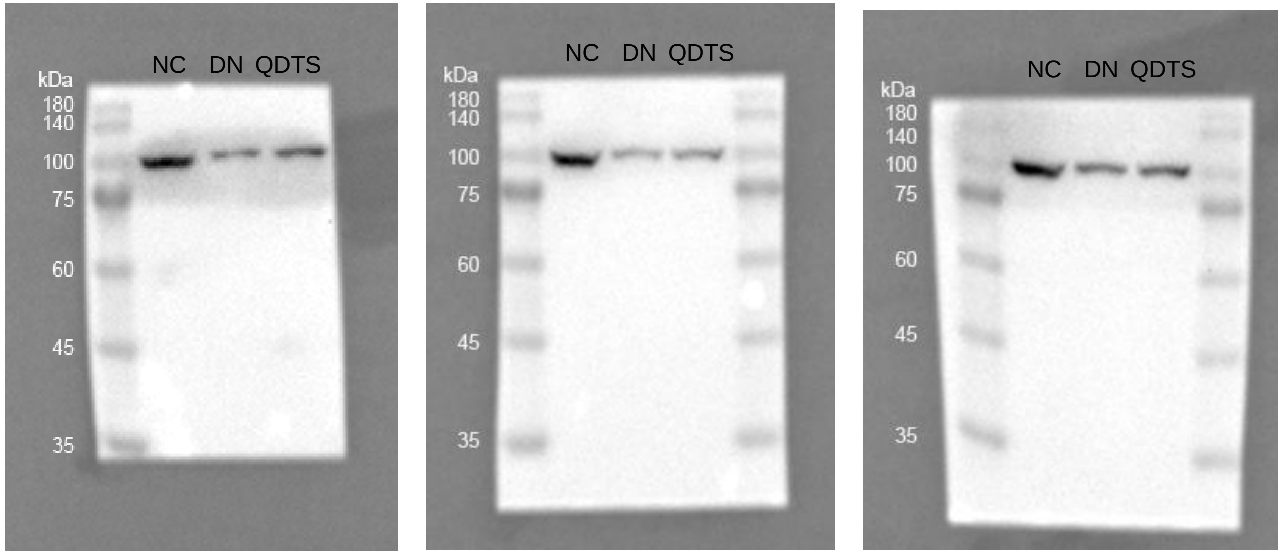

Figure S3(8B)

mice kidney tissue
GAPDH(36 kDa)

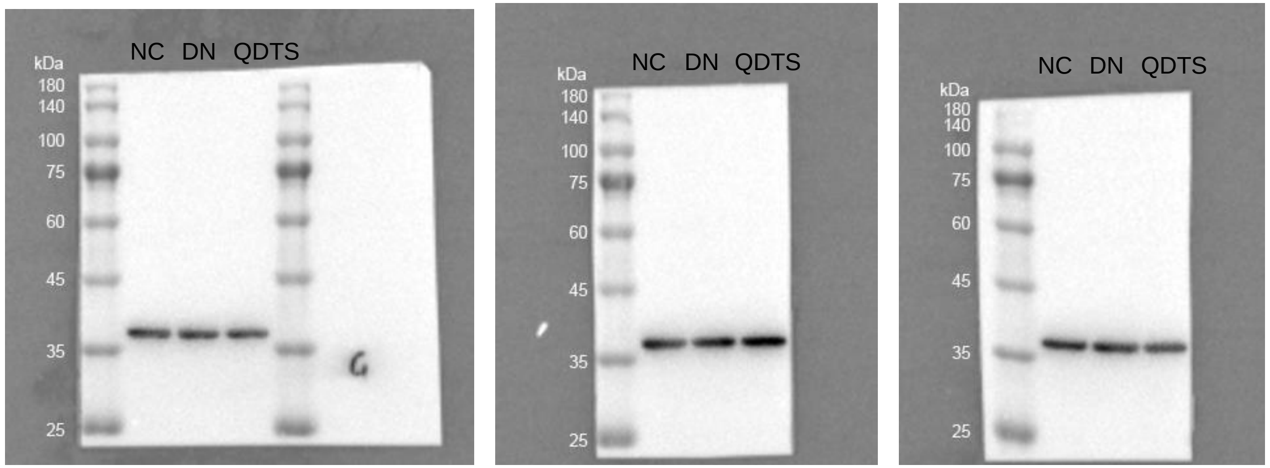

Figure S4(8C)

MPC5 cells
Nephrin(135 kDa)

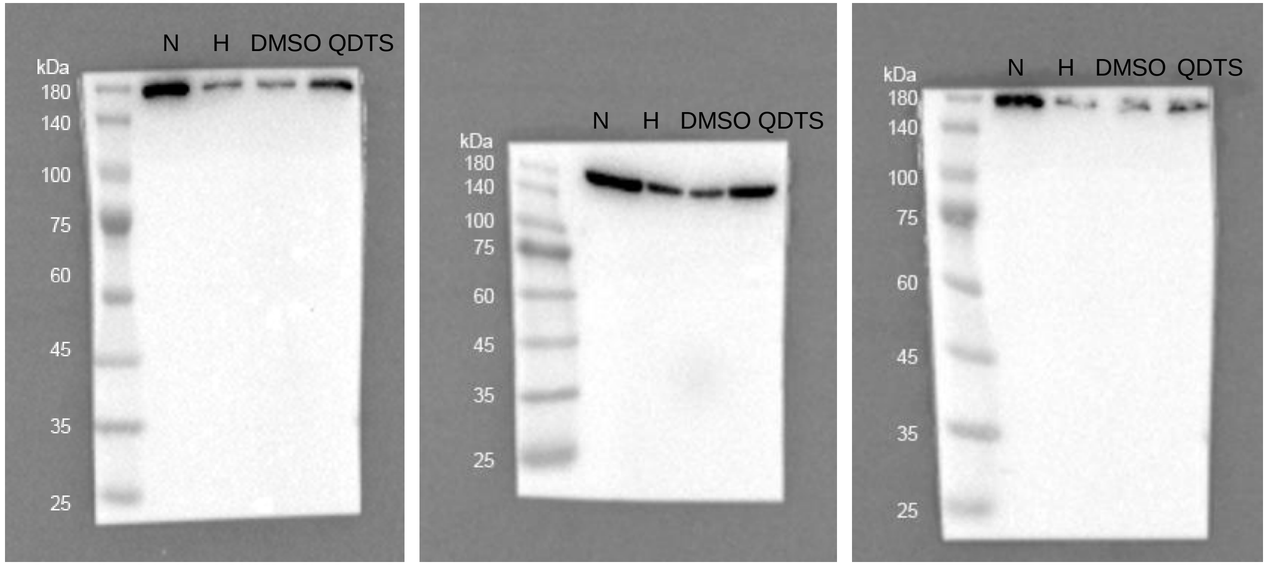

Figure S5(8C)

MPC5 cells
Synaptopodin(99 kDa)

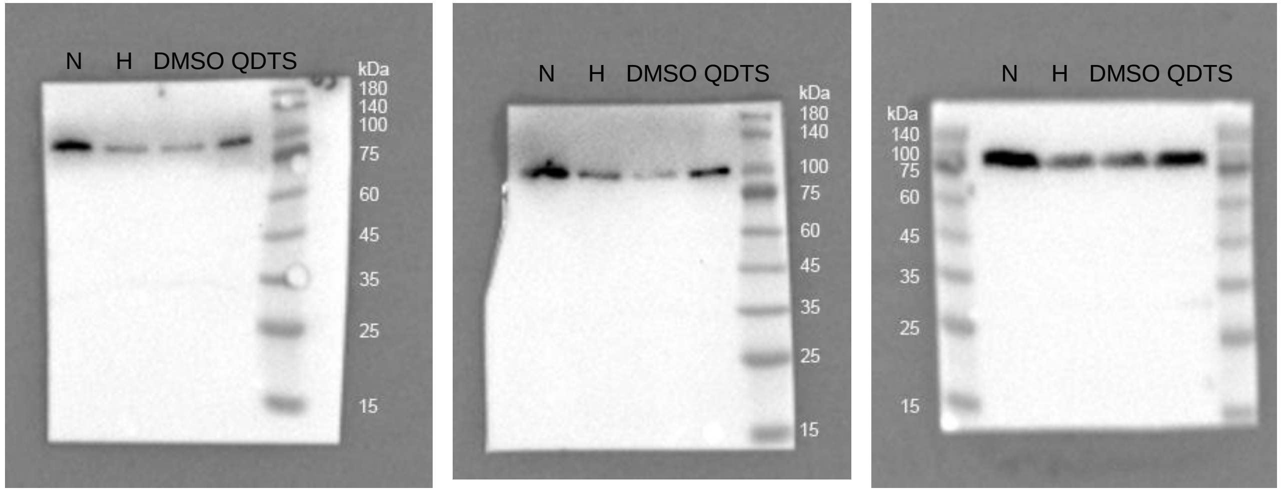

Figure S6(8C)

MPC5 cells
GAPDH(36 kDa)

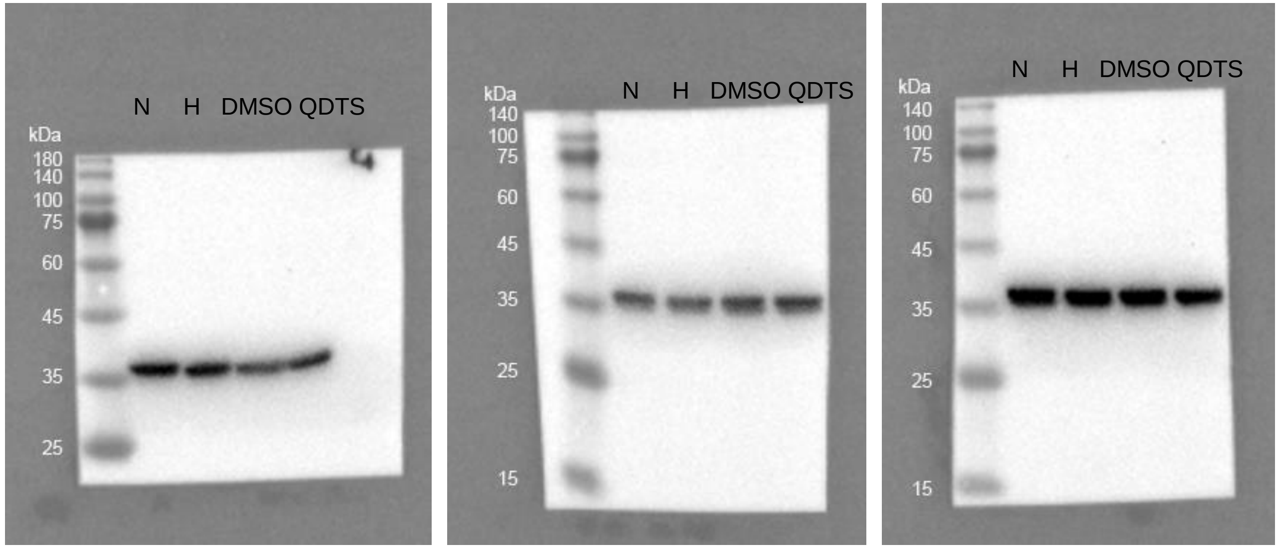

Figure S7(9C)

mice kidney tissue
Phospho-PI3K(80 kDa)

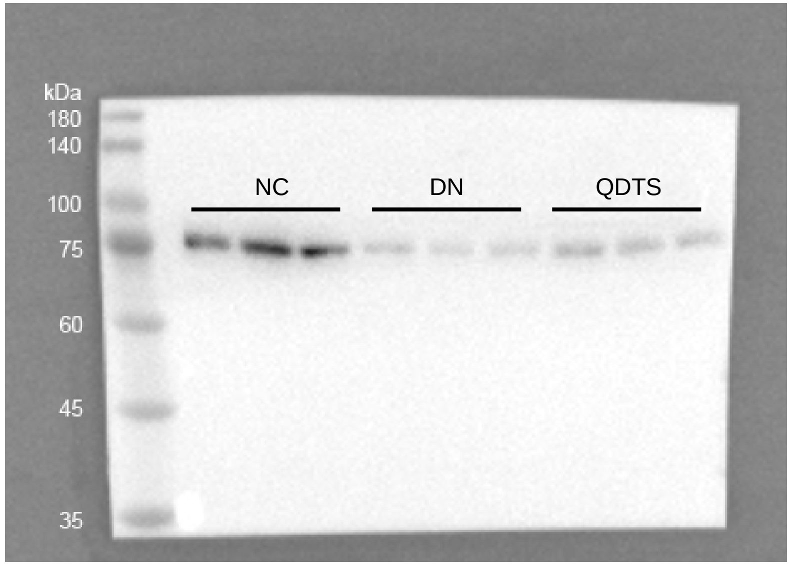

Figure S8(9C)

mice kidney tissue
Phospho-AKT(60 kDa)

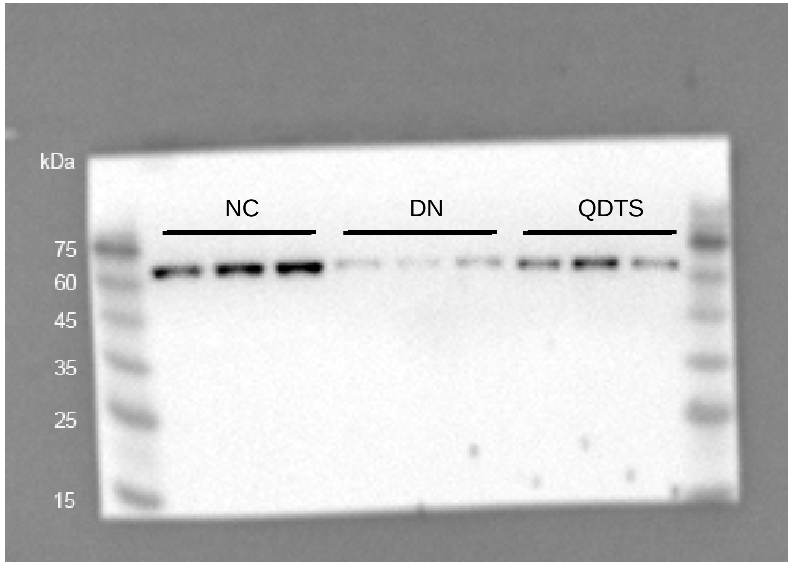

Figure S9(9C)

mice kidney tissue
JUN(36 kDa)

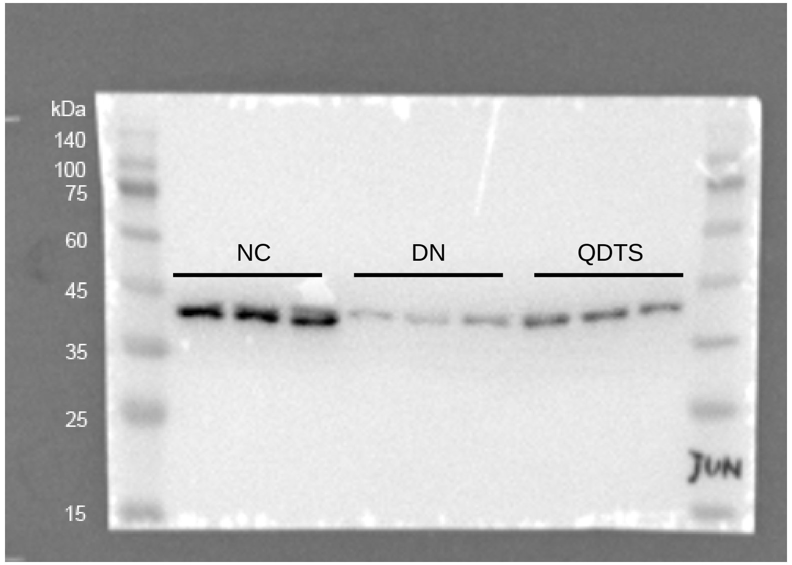

Figure S10(9C)

mice kidney tissue
Nephrin(135 kDa)

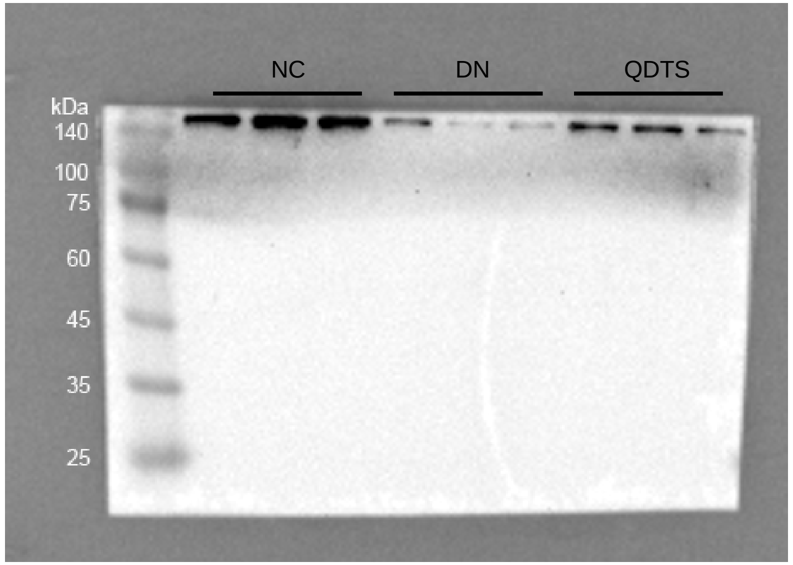

Figure S11(9C)

mice kidney tissue
Synaptopodin(99 kDa)

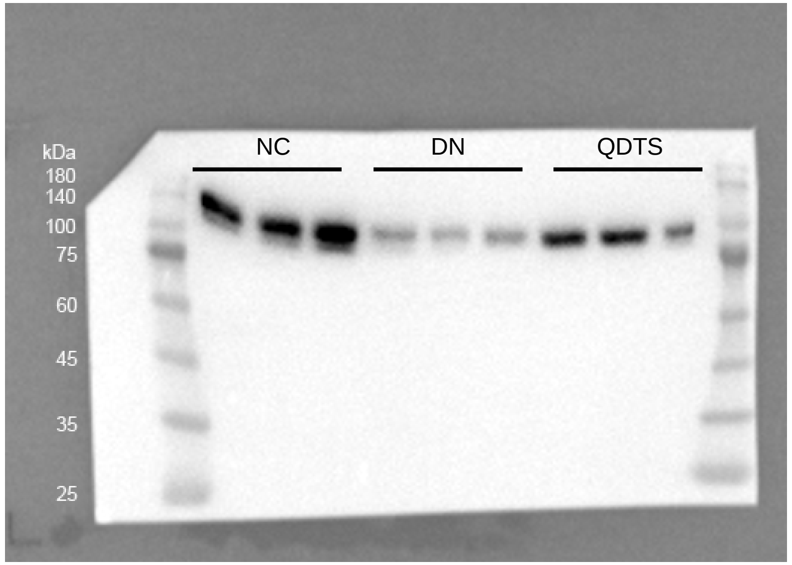

Figure S12(9C)

mice kidney tissue
GAPDH(37 kDa)

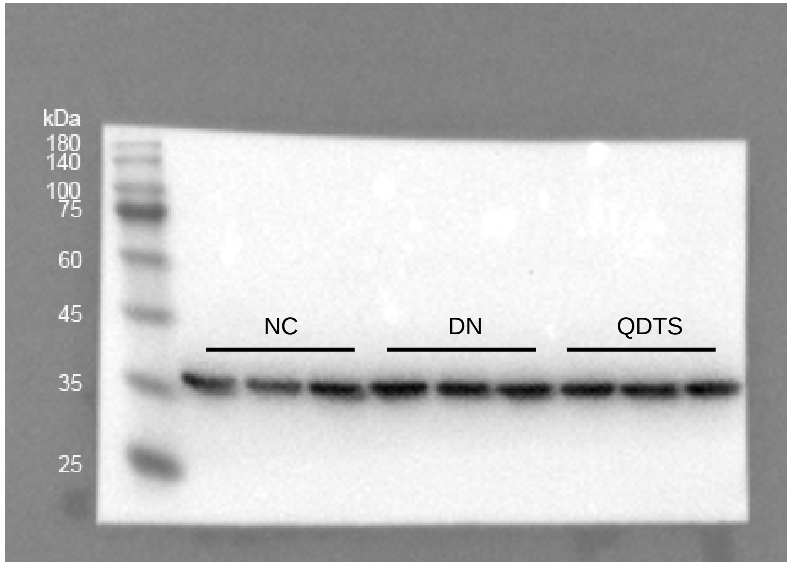

Figure S13(9E)

MPC5 cells
Phospho-PI3K(80 kDa)

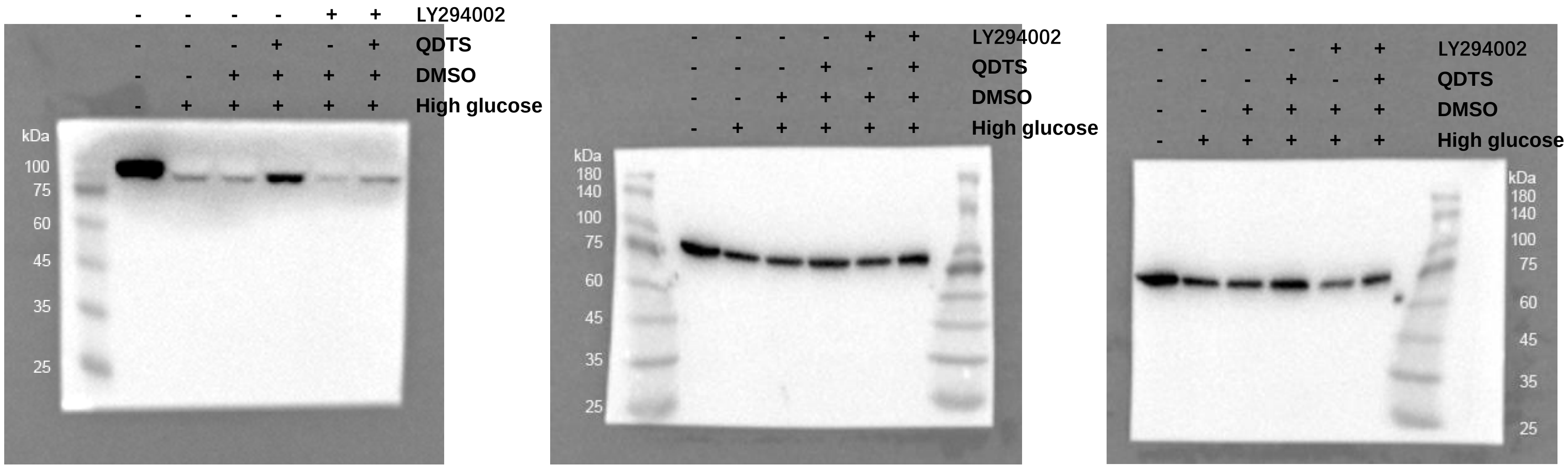

Figure S14(9E)

MPC5 cells
Phospho-AKT(60 kDa)

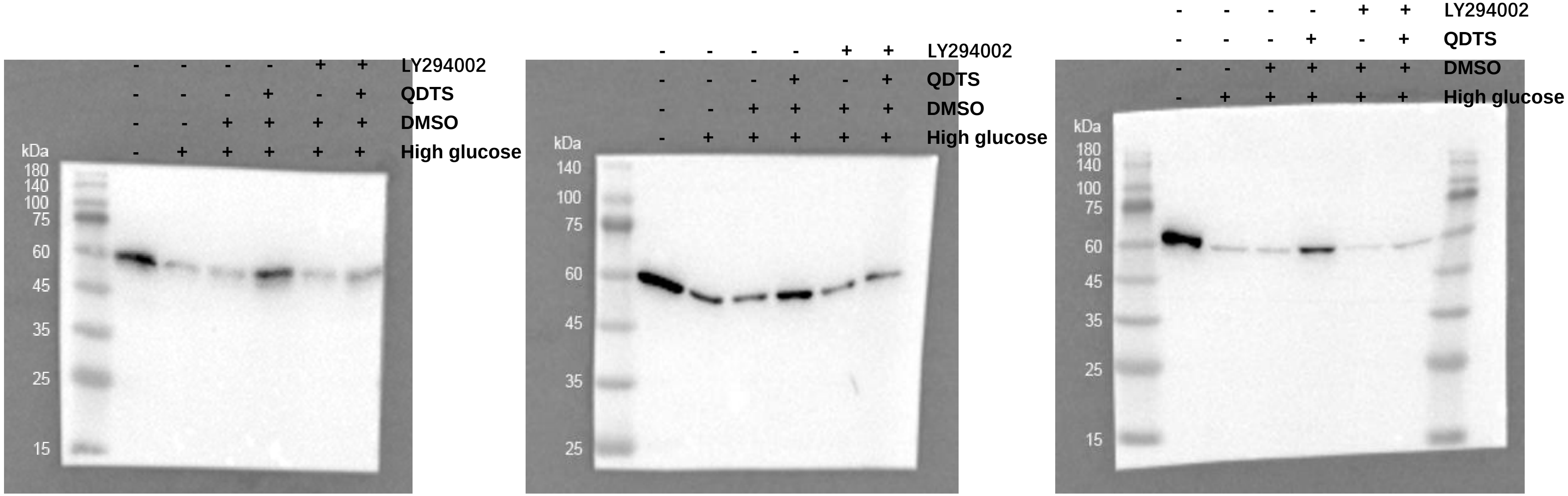

Figure S15(9E)

MPC5 cells
JUN(36 kDa)

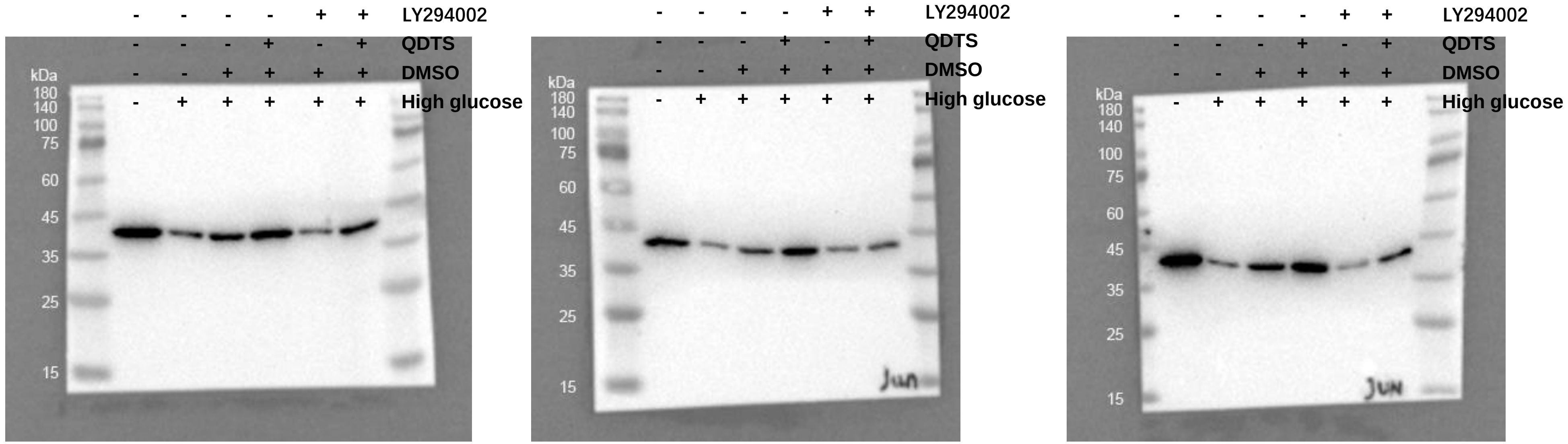

Figure S16(9E)

MPC5 cells
Nephrin(135 kDa)

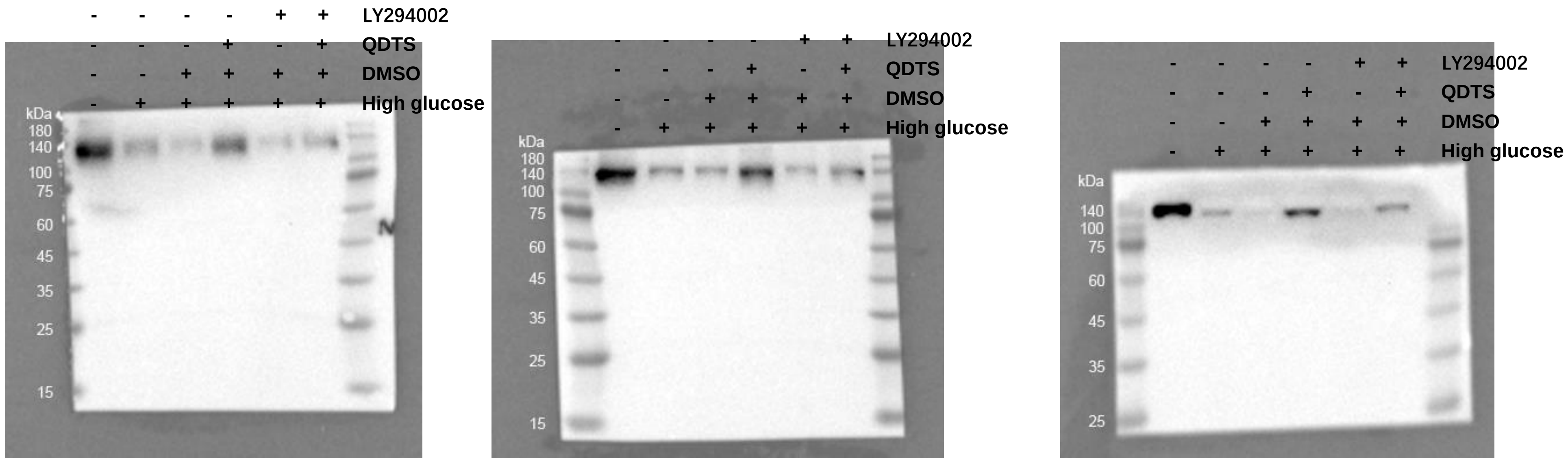

Figure S17(9E)

MPC5 cells
Synaptopodin(99 kDa)

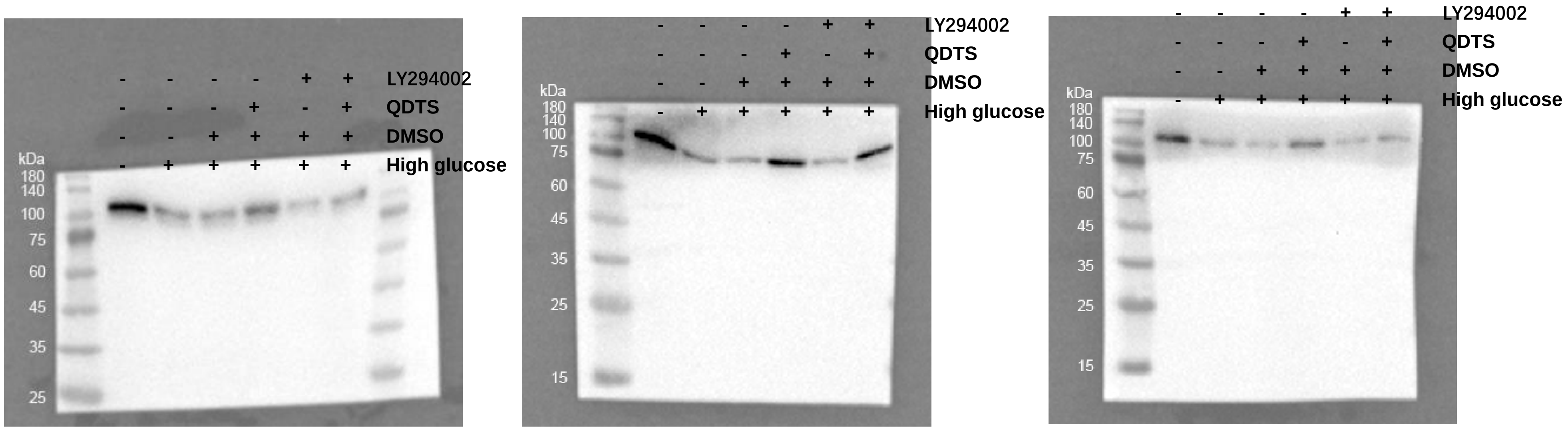

Figure S18(9E)

MPC5 cells
GAPDH(37 kDa)

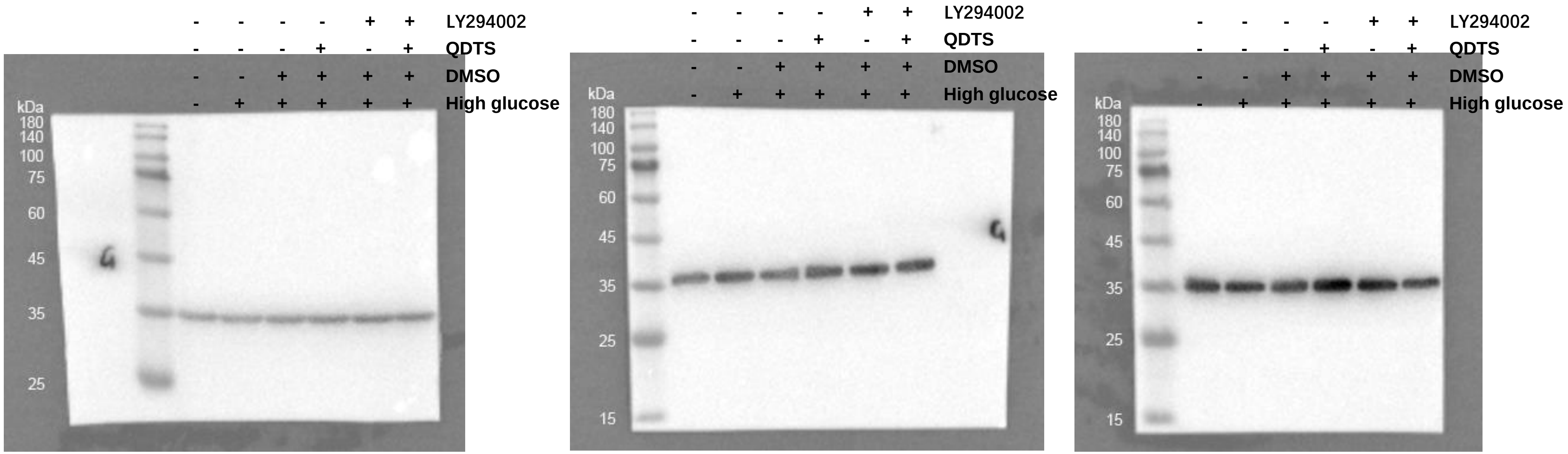

Supplement: Multimedia component 1 [file mmc1.pdf]
